# Machine learning-aided design of composite mycotoxin detoxifier material for animal feed

**DOI:** 10.1038/s41598-022-08410-x

**Published:** 2022-03-22

**Authors:** Giulia Lo Dico, Siska Croubels, Verónica Carcelén, Maciej Haranczyk

**Affiliations:** 1 IMDEA Materials Institute, C/Eric Kandel 2, 28906 Getafe, Madrid Spain; 2 Department of Materials Science and Engineering, Universidad Carlos III de Madrid, Avda. de la Universidad, 30, 28911 Leganés, Madrid Spain; 3 Tolsa Group, Carretera de Madrid a Rivas Jarama, 35, 28041 Madrid, Spain; 4 Department of Pathobiology, Pharmacology and Zoological Medicine, Faculty of Veterinary Medicine, Ghent University, Salisburylaan 133, 9820 Merelbeke, Belgium

**Keywords:** Diagnostic markers, Design, synthesis and processing, Cheminformatics

## Abstract

The development of food and feed additives involves the design of materials with specific properties that enable the desired function while minimizing the adverse effects related with their interference with the concurrent complex biochemistry of the living organisms. Often, the development process is heavily dependent on costly and time-consuming in vitro and in vivo experiments. Herein, we present an approach to design clay-based composite materials for mycotoxin removal from animal feed. The approach can accommodate various material compositions and different toxin molecules. With application of machine learning trained on in vitro results of mycotoxin adsorption–desorption in the gastrointestinal tract, we have searched the space of possible composite material compositions to identify formulations with high removal capacity and gaining insights into their mode of action. An in vivo toxicokinetic study, based on the detection of biomarkers for mycotoxin-exposure in broilers, validated our findings by observing a significant reduction in systemic exposure to the challenging to be removed mycotoxin, i.e., deoxynivalenol (DON), when the optimal detoxifier is administrated to the animals. A mean reduction of 32% in the area under the plasma concentration–time curve of DON-sulphate was seen in the DON + detoxifier group compared to the DON group (*P* = 0.010).

## Introduction

The growing world population and its impact on the environment creates a need for advanced agriculture technologies and products, for example, highly target-specific animal feed additives to improve the condition and growth of livestock^1–3^. Mycotoxin detoxifiers (MDTs) are an example of such additives^4,5^. Mycotoxins are toxic secondary metabolites typically produced by fungi growing in food and animal feed commodities^6,7^, and exposure to these contaminants may result in death or disease^8,9^. The maximum levels of some of the most highly prevalent mycotoxins in animal feed are already controlled guidance values or recommendations, rendering the need for mitigation strategies, one of which is mycotoxin capture by detoxifier feed additives^10^. The global market of the latter is projected to grow at the rate of 3.1% from 2020, and reaching a value of USD 3.1 billion by 2027^11–14^.

A typical mode of action of MDTs involves either forming bulky non-absorbable complexes with mycotoxins in the gastrointestinal tract, hence reducing their oral bioavailability, or promoting their degradation into non-toxic metabolites by bio-transforming agents, such as bacteria or enzymes^1,15,16^. Adsorbents capture mycotoxins which are delivered through the gastrointestinal tract of the animals with their feed, and the mycotoxin-MDT complex is eliminated with feces thus minimizing the absorption in the blood stream^17^. Among the mycotoxin-binding agents (binders), inorganic porous materials such as clays minerals are recognized effective especially for sequestering of aflatoxin B1 (AFB1)^18,19^. However, the specific mode of action strongly depends on the affinity between clay and mycotoxins, which is a function of multiple variables representing the structure and properties of the mycotoxins, the adsorbing materials as well as the process, e.g. inclusion rate of MDTs^18^. For example, montmorillonites are commonly used for aflatoxins binding by creating complexes with the exchangeable cations, while stevensites exploit their larger surface area for efficient entrapping of ochratoxin A (OTA) and zearalenone (ZEN)^20,21^. Material processing strategies such as the preparation of organo-aluminosilicates have made improvement in the uptake capability of ZEN, OTA and T-2 toxin (T2)^22^. Similarly, other porous materials such as activated charcoal (AC) have been recognized as strong adsorbents for several mycotoxins, including deoxynivalenol (DON)^23^. However, the required AC doses necessary for a significant detoxification leads to sequestrating of essential micronutrients such as vitamins or minerals^24^. Composite materials based on a mixture of the above may offer to capture and remove multiple large number of mycotoxins while minimizing the interference effect. Besides the regulated mycotoxins, yet-unregulated and emerging mycotoxins occur frequently in agricultural products^25^, thus it is desired that the detoxifier design should address a wide variety of toxic fungal metabolites without compromising the animal health^26^.

The development of advanced MDTs additives represents a multidisciplinary endeavor. It involves the material design of the additive to optimize selective toxin adsorption while relying on the fields of biochemistry, biology and veterinary medicine to understand the mechanisms of action and properly assess the impact, efficacy and potential side effects of the additive. In vitro experiments imitating the gastrointestinal environment may be used for the preliminary assessment of promising MDT, and are recommended by, for example, the European Food Safety Authority (EFSA)^27^. However, low fidelity of reproduction of the complexity of the true animal’s digestive system by such models requires in vivo experiments for definitive performance evaluation, one of which are strategies based on biomarker for exposure detection^28–31^.

Interestingly, if the interdisciplinary MDTs development were to rely on the state of the art of the respective disciplines, it would have to combine seemingly conflicting research and development strategies. In the case of material science, (semi)automated high-throughput synthesis and characterization, as well as computational screening approaches are allowing to generate and assess the performance of an unprecedented number of high performing materials, including porous materials^32–34^. On the contrary, the research involving living animals is guided by the principle of the “3Rs”, i.e. replacement, reduction and refinement when animals are used for scientific purposes to improve animal welfare and to minimize the environmental impact^35,36^. Herein, we present an approach that addresses this issue and allows for screening of vast number of material formulations while incorporating the animal-derived models for efficacy testing. Specifically, we have built machine learning (ML) models that incorporate three distinctive factors underlying the detoxifier performance, i.e. the material formulation, the chemical structure of the targeted toxin and the process in which a MDT is applied. The models being trained using an extensive set of in vitro experiments are used as surrogates of real experiments for the exploration of promising MDTs. In this study, we aim to demonstrate three-fold applications of our approach (1) in the identification of high performing formulations for the regulated toxins, (2) in predicting detoxification of a wide set of yet-unregulated mycotoxins, and (3) in gaining insights into the in vitro detoxification mode of action through model feature importance analysis. Finally, biomarker detection-based in vivo validation of the material identified in (1) is demonstrated in a challenging DON detoxification trial in broiler chickens.

## Results and discussion

### Predictive models and approach overview

The performance of MDTs could be most definitely determined on the basis of long-term studies assessing the growth and health of living animals^37,38^. It is, however, impractical and costly to implement such studies in the context of MDT material design. Instead, the performance is measured by either short-term in vivo determination of toxin biomarkers for exposure after single bolus administration, or by much faster in vitro experiments^27^, which are set up to mimic the toxin adsorption–desorption cycle experienced by the detoxifier passing through the gastrointestinal tract^39^ (see Supplementary Sect. S1 for details). The performance metrics corresponding to the latter approach, Ads(%) and Eff(%), are the percentages of the initial toxin level actively adsorbed on the MDT in the modeled gut experiment and removed from the digestive tract with the MDT, respectively.

**Figure 1:**
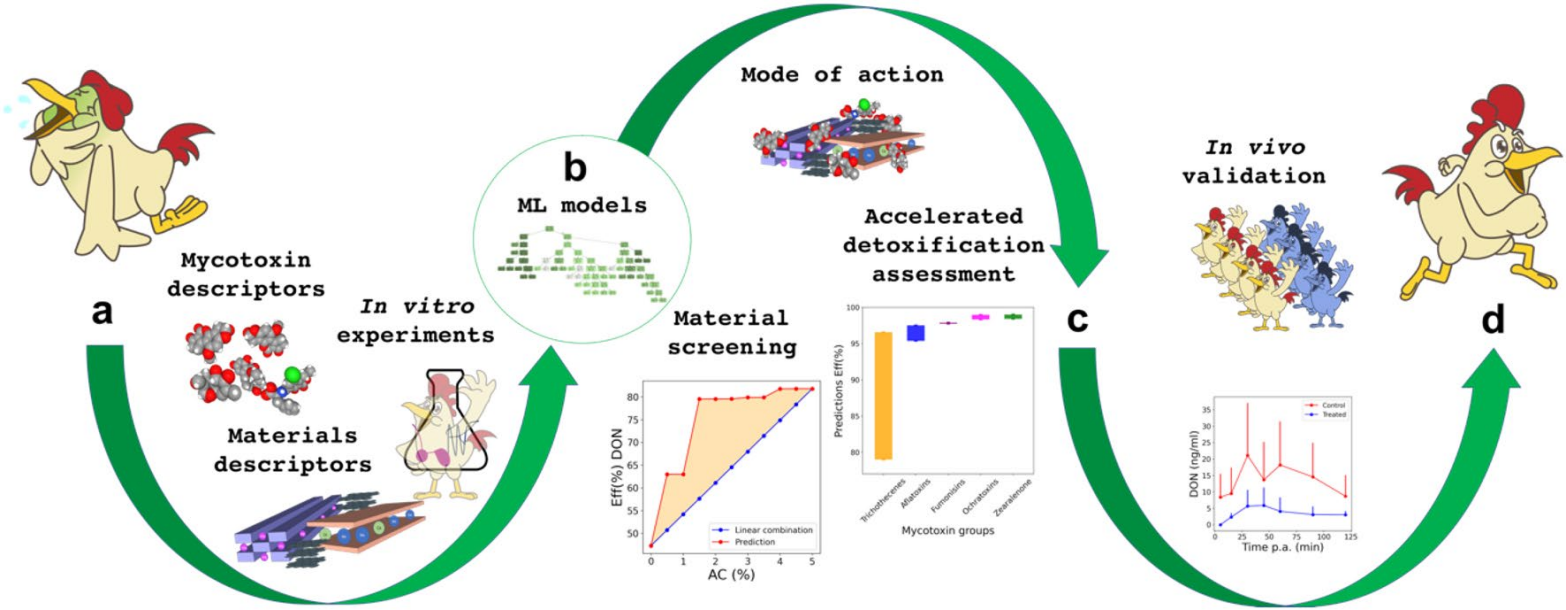
Workflow representation starting from selection of feature vector space (**a**) which describes the in vitro adsorption and efficiency (model targets). Machine learning models (**b**) trained on in vitro dataset providing tools for material screening, wide in vitro detoxification assessment, and mode of action capturing (**c**). In vivo validation of the findings extracted by our approach (**d**).

The sequestering capability of MDT is the function of the composition of the MDT material itself, the chemical structure of the targeted toxin as well as a number of parameters defining the application process such as dose^40^. In the applications thus far, the identification of the optimum of this function has been tedious and costly due to a large parameters space and heavy reliance on experimentation determination of Ads(%) and Eff(%). Herein, we introduce an approach, which workflow is summarized in Fig. 1, that provided to build surrogate models of Ads(%) and Eff(%) allowing for a quick identification of powerful MDTs.

In our approach, the information on MDT performance is a function of descriptor space which encodes clay materials with experimentally-determined physico-chemical features, mycotoxins with molecular descriptors, and the modeled in vitro experiments with five setting parameters (Fig. 1a). Then, we develop random forest regressor models (RF) predicting both Ads(%) (denoted RFads) and Eff(%) (denoted RFeff) using 84 experimental data points (Fig. 1b). The data distribution shown in Supplementary Fig. S1, demonstrates the low correlation between in vitro adsorption, desorption and efficiency outcomes contained in the dataset. The dataset comprises 15 diverse toxin-detoxifier materials (MDTs), i.e., 10 natural clays and 5 clay-based composites in which formulation the organic component is activated charcoal (AC) (see method section for details). The two independent machine learning models exhibit high predictive performances with R^2^ score of 0.92 and 0.96 for RFads and RFeff, respectively (details in method section). Upon the positive assessment of the models, three main contributions were investigated, i.e., screening of optimal solutions, wide in vitro detoxification assessment, and mode of action capturing (Fig. 1c). Finally, biomarker detection-based in vivo trial in broiler chickens was performed as proof-of-concept to validate our approach, hence, the sequestration power of the top powerful MDT towards the sequestration of a mycotoxin (DON) selected among the most challenging to be mitigated (Fig. 1d).

**Figure 2:**
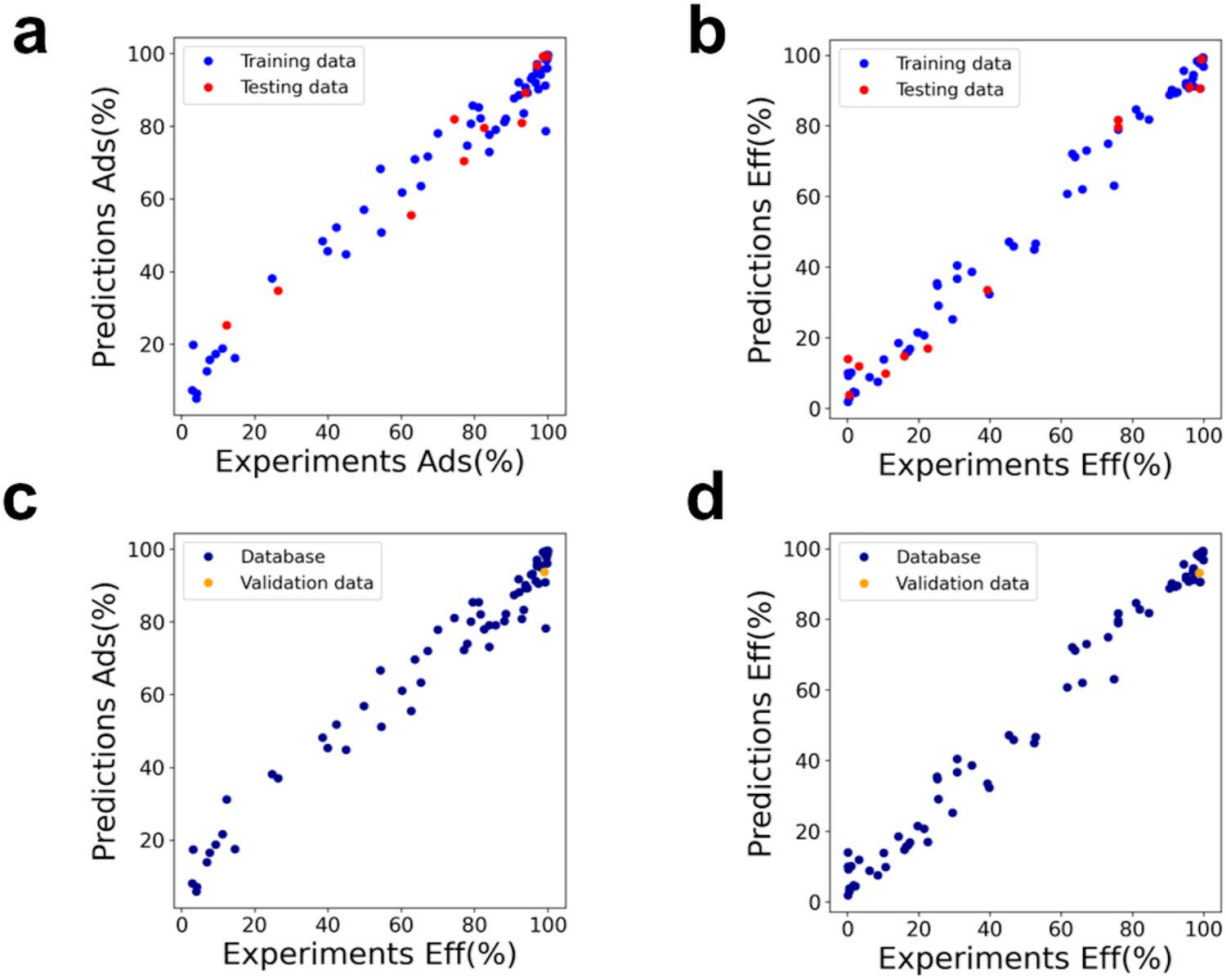
Graphical assessment of RFads (**a**) and RFeff (**b**) of 6 mycotoxins by 15 MDTs under different experimental conditions. Validation of RFads (**c**) and RFeff (**d**) toward one yet-unregulated toxin (DAS).

Figure 2a, b provide the graphical models assessment and Supplementary Fig. S2 summarizes the associated mean absolute error distributions given by the model, which exhibit apparent absence of systematic errors. The models were further validated by predicting and testing the Ads(%) and Eff(%) of a composite material, which incorporates 5% AC, toward an out-of-trainset trichothecene toxin, i.e. diacetoxyscirpenol (DAS).

Our models are able to capture and quantify the correlation between the differences among mycotoxin chemical structures and the physico-chemical features of MDT providing extremely accurate predictions (Fig. 2c, d) as well as reproducing the experimentally obtained ranking: DON > DAS > T2. Specifically, the chemical structures of two trichothecenes contained into the dataset (DON, T2) were compared with the chemical structure of DAS and shown in Supplementary Fig. S3. T2 joins two acetoxy and one methylbutanoate group in the DON structure, while DAS only shares with T2 the acetoxy groups. The corresponding slight variations of mycotoxin molecular properties complicate the human interpretability, while the appreciable model accuracy allows screening MDT materials toward the removal of yet-unregulated mycotoxins enabling fast future detoxification assessment in case of emerging contaminants.

### Mode of action analysis

**Figure 3:**
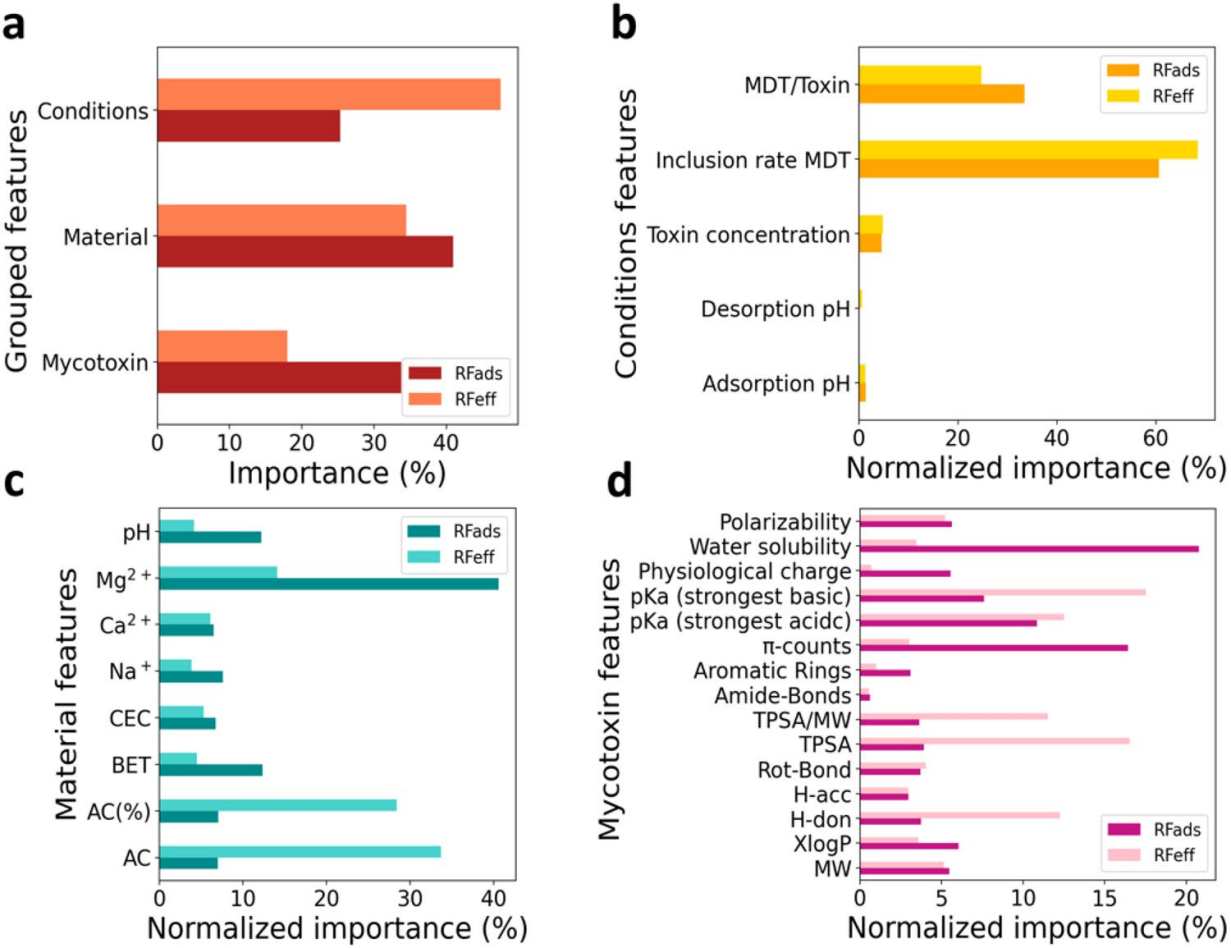
Summed importance score of the groups of features outlined in Table 2, extracted by RFads and RFeff (**a**). Normalized individual contribution corresponding to in vitro experimental conditions (**b**) materials (**c**) and mycotoxin (**d**) group of features.

Feature importance analysis of the ML models provides insights into the main descriptors affecting the detoxification and, indirectly, the mode of action of MDT in in vitro scenarios. Figure S4 depicted the feature importance scores extracted by RFads and RFeff. We analyzed separately the impact of each group of descriptors by summing the corresponding scores assigned to the descriptors belonging to its group (Fig. 3a). We then normalized the individual contributions from 0 to 100% where the 100% is assigned to the summed importance score of the experimental conditions (Fig. 3b), materials (Fig. 3c) and mycotoxin (Fig. 3d) group of features. The main contribution to Ads(%) comes from material descriptors while Eff(%) is mostly determined by the conditions of the in vitro experiments (Fig. 3a), in which the inclusion rate and the MDT/toxin ratio possess the highest score (Fig. 3b). The high contribution of the latter enables our models to screen possible dosages identifying the recommended amount of MDT administration for specific contamination levels.

The mode of action is dominated by the unequivocal heavy contribution of Mg^2+^ (Fig. 3c), which suggests that the adsorption mechanism involves substitutions of the exchangeable cations and chelate formations. However, the importance score distribution indicates that a multifunction of diverse interactions e.g. hydrogen bonds, Van der Waals, electrostatic and hydrophobic interaction are involved. The type of AC, i.e. high or low surface area form, and its content in composite formulation (AC(%)) are dominating the RFeff. Water solubility and π-counts are the main mycotoxin descriptors (Fig. 3d) involved during the adsorption step, suggesting that the primary interaction is entropically promoted by hydrophobic and π effects which involves replacing of solute–solvent and surface-solvent bonds by solute-surface and solvent–solvent bonds. Those are weak interactions which prevent a robust binding with the detoxifier along the whole digestive tract and lose importance when the desorption step is considered. The high contribution in RFeff of mycotoxin’s pKa, the topological polar surface area, and amount of hydrogen-bond donors indicates that electrostatic interactions with MDT surface are responsible for ensuring an effective sequestration during the modelled gastrointestinal tract.

### Towards optimal MDT designs

The experimental in vitro performance of three different pure clays against the uptake of the six main mycotoxins included in the dataset (Supplementary Fig. S5) reveals the clay’s selectivity towards specific mycotoxins, e.g. sepiolite and montmorillonite exhibit a stronger affinity towards DON than smectite. An effective uptake of multiple mycotoxins can be achieved by incorporating various components in the MDT design. The latter can be achieved by the optimal formulation in which the components positively cooperate increasing the simultaneous binding of diverse mycotoxins.

**Figure 4:**
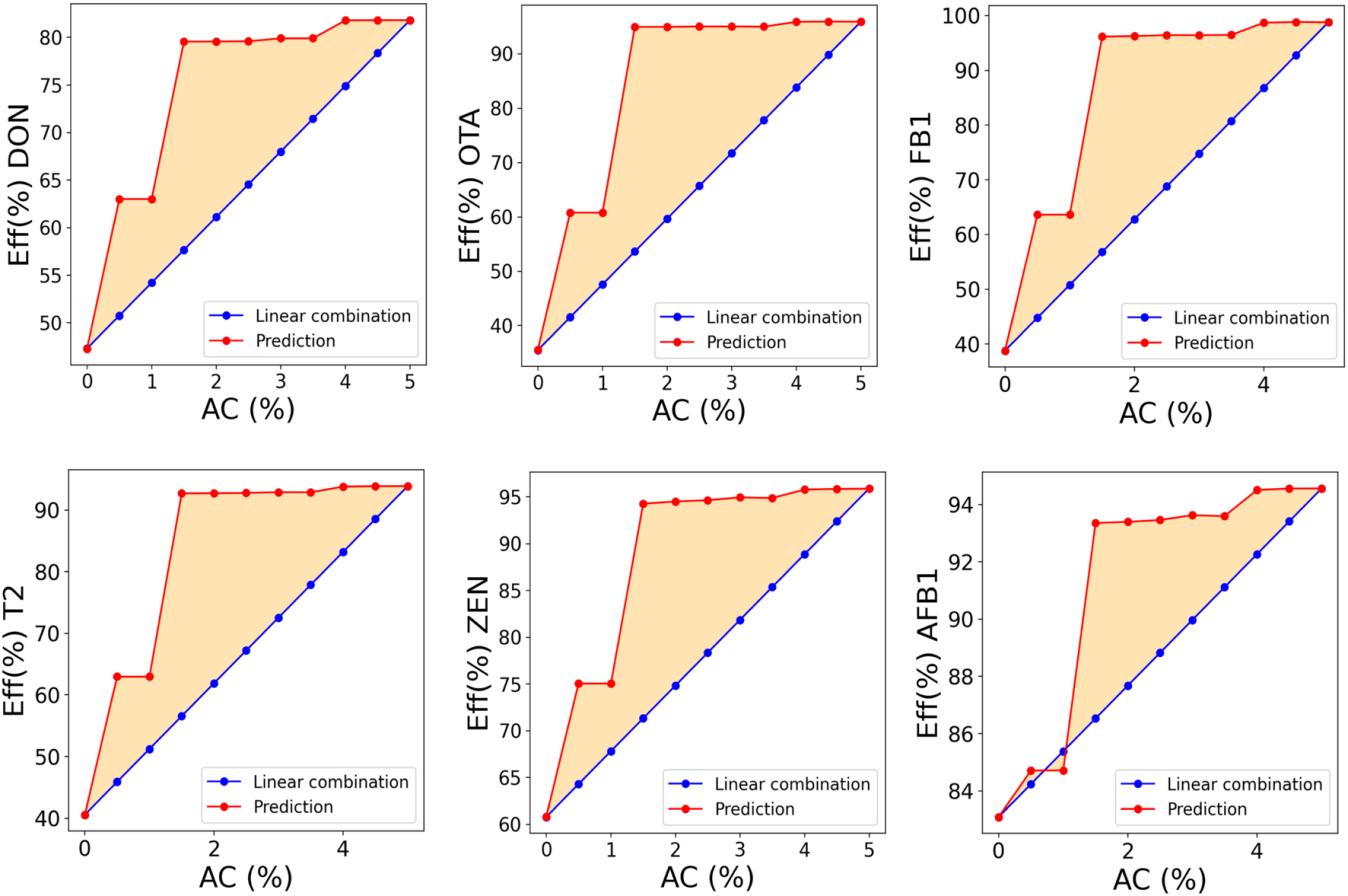
Synergy capturing by RF predicting efficiency for a series of hybrids of sepiolite-montmorillonite-charcoal in which the sepiolite-montmorillonite ratio was fixed to 1/4. The orange area is assigned to the positive synergistic effect. The experimental settings for the uptake of DON, OTA, T2, FB1 and ZEN were fixed to 2 kg/t of inclusion rate of MDT, 2 µg/ml of toxin concentration. The pH during the adsorption experiment was fixed to 3 while the desorption pH was 6.5.

In order to identify MDTs with high performance, expressed by RFeff, we screened a number of hybrid formulations which incorporate clays with activate carbon. The latter, being highly porous may cause the sequestration of micronutrients prejudicing the animal health. Together with the highest efficiency we addressed the optimization looking for those formulations in which each component positively cooperates with the others in order to reduce the amount of activated carbon need to reach the desired performance, hence limiting the interferences with micronutrients. Thus, we proposed a function to estimate the synergic effect between components and it is expressed by Eq. (4). The synergy was estimated as the difference between the predicted Eff(%) of hypothetical formulations and the linear combination of Eff(%) associated with a mixture of pure components (details in “Methods” section). The descriptors of hypothetical materials were calculated as a linear combination of the pristine descriptors (see “Methods” section for details). The capability of RFeff in estimating the synergistic effect was assessed predicting the Eff(%) of a number of sepiolite-montmorillonite mixtures. Supplementary Fig. S6 demonstrates the positive cooperation between the two explored clays (sepiolite and montmorillonite). One of the formulations that expressed the highest synergy, composed of sepiolite/montmorillonite in ratio 1:4, was prepared and tested for an in vitro detoxification of ZEN. The experiments are close to the prediction of Eff(%) within the corresponding error bars, i.e., the model predicted an Eff(%) of 58.3 ± 4.89 while the experimented Eff(%) was 67.5 ± 5.7. The following exploration of possible composite material compositions have additionally incorporated high surface AC as a third component. In this case, the synergy was defined as the difference between the predicted outcomes of hypothetical formulations and the linear combination of the outcomes of composite formulation with the highest amount of AC (fixed at 5%) (details in “Methods” section). All the possible sepiolite-montmorillonite-charcoal combinations were screened by RFeff and the optimal formulations were identified in those incorporating 20–40% of sepiolite and at least AC 1.5%. Figure 4 enables synergy exploration of optimal formulations during the successful removal of the main regulated mycotoxins. We observed a plateau starting from those hybrids which incorporates sepiolite-montmorillonite in ratios of 1/4 and 1.5% of AC. The latter formulation also express the highest synergy. However, the composite sepiolite-montmorillonite in ratio 1:4 and 2% of AC (SEP/MONT/AC) was selected as the top performing MDT due to easier material preparation scale-up.

### Machine learning-aided assessment of the identified SEP/MONT/AC composite

**Figure 5:**
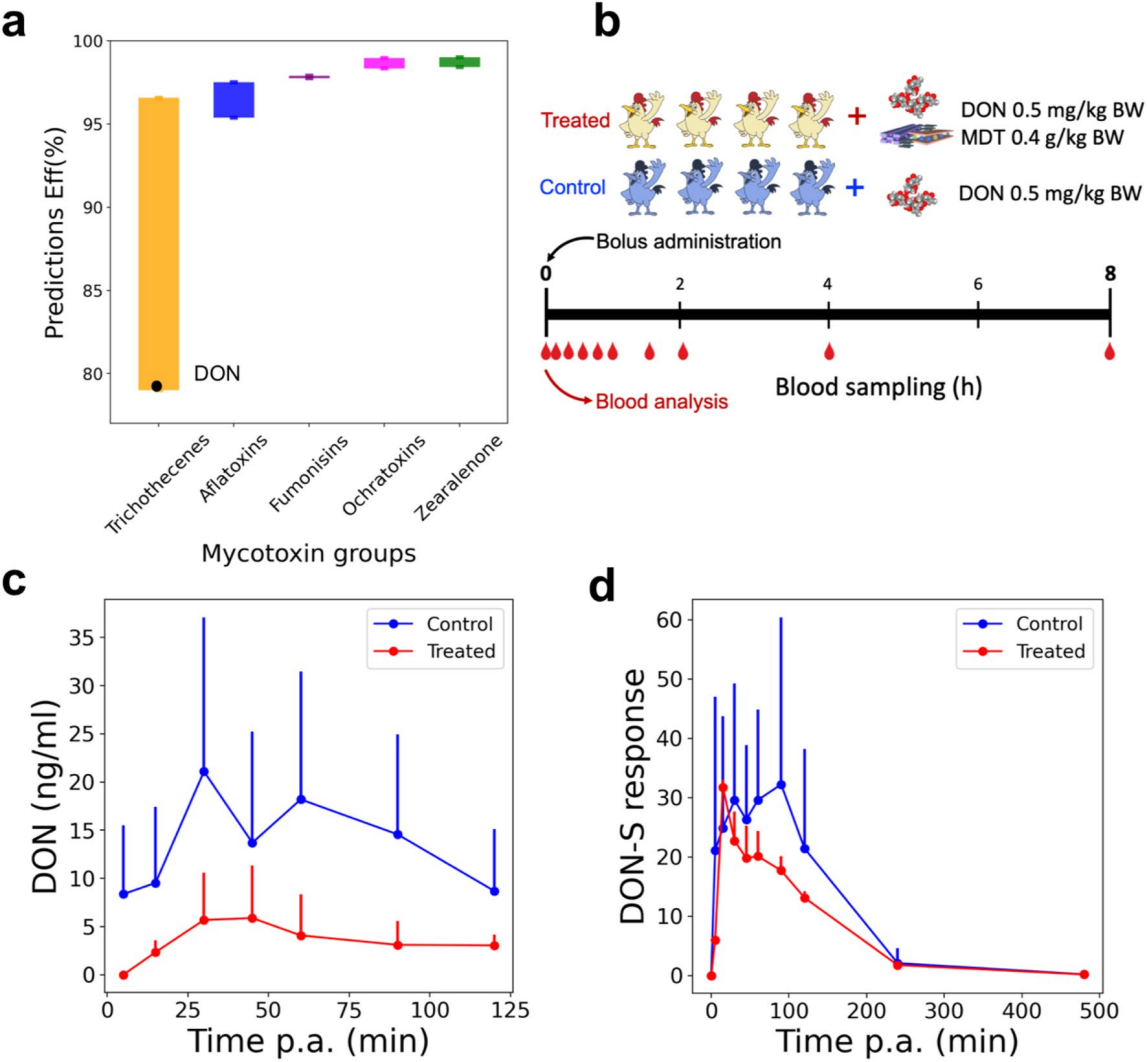
Response of predicted in vitro efficiency of SEP/MONT/AC towards the removal of the explored mycotoxin groups (**a**). The predictions were obtained by RF fixing the experimental setting to 2 kg/t of inclusion rate of SEP/MONT/AC, 2 µg/ml of toxin concentration, adsorption and desorption pH to 3 and 6.5, respectively. Graphical representation of in vivo design of experiment validating the detoxification of DON by SEP/MONT/AC detoxifier (**b**). Mean plasma concentration and standard deviation of DON after single oral bolus administration of DON alone (0.5 mg/kg BW) and DON in combination with SEP/MONT/AC (0.4 g/kg BW) to 8 broiler chickens (**c**). Mean response and standard deviation of deoxynivalenol-sulphate (DON-S) in plasma after single oral bolus administration of DON alone (0.5 mg/kg BW) and DON in combination with SEP/MONT/AC (0.4 g/kg BW) to 8 broiler chickens (**d**).

Our computer-aided approach enables fast screening of emerging contaminants limiting the need for animal tests, hence following the 3Rs principles. Moreover, the practical implementation of RFeff in screening diverse inclusion rates and toxin concentration values recommends the optimal to be administrated MDT dosage under specific contamination levels. The high accuracy of RFeff model in predicting the detoxification of out-of-dataset molecules (Fig. 2d) was employed to assess SEP/MONT/AC against the removal of a large set of yet-to-be-regulated mycotoxins (Supplementary Table S1). Supplementary Figure S7 summarizes the individual predicted efficiencies. Figure 5a reveals the reliable detoxification of SEP/MONT/AC achieving an efficiency of at least 94% in all the explored toxin groups, except for the trichothecenes, which are the most heterogeneous group of molecules. DON was found to be the most challenging-to-be-removed toxin by SEP/MONT/AC, thus it was selected to validate our findings through an in vivo test.

### In vivo validation assessment

We further evaluated the sequestration power of the identified SEP/MONT/AC composite through in vivo experiments carried out by a biomarker detection of DON^27^, albeit after a single bolus toxin exposure. We performed the in vivo trial to validate our findings by observing the DON relative oral bioavailability when the detoxifier is administrated to broiler chickens. The design of in vivo trial is schematized in Fig. 5b and detailed in method section and Supplementary Sect. S1. The broiler chickens were equally divided into treated (0.5 mg/kg BW of DON and 0.4 g/kg BW of SEP/MONT/AC, inclusion rate of 4 kg/t) and control (0.5 mg/kg BW of DON) groups, their repetitive blood analysis was performed recording the mean plasma concentration–time curve of DON. Figure 5c displays a significant reduction in DON oral availability after oral bolus administration of DON, whether combined with the detoxifier (treated group) compared to control group. Since in many samples DON could not be detected, or the concentration was below the limit of quantification of 1 ng/ml, no accurate toxicokinetic modeling could be performed for DON. Therefore, the metabolite DON-sulphate (DON-S) was used to perform the modeling. This metabolite is a major phase II biotransformation product of DON and hence a suitable biomarker for DON exposure in broiler chickens^41–43^. Figure 5d demonstrates the mean plasma response-time curve of the metabolite DON-S after oral bolus administration of DON, whether or not combined with the mycotoxin detoxifier to 8 broiler chickens. Table 1 summarizes the results of the most important toxicokinetic parameters of the metabolite DON-S after oral bolus administration of DON to 8 broiler chickens, whether or not combined with the detoxifier. A significantly lower systemic exposure of DON-S, expressed as AUC_0–∞_, was seen for the DON + detoxifier group compared to the DON group (*P* = 0.010). Also, a statistically significant lower Cmax for DON-S was seen for the DON + detoxifier group compared to the DON group (*P* = 0.013). Furthermore, the relative oral bioavailability of DON-S in the DON + detoxifier group compared to the DON group was only 67.8%. This shows a relevant reduction in in vivo systemic exposure to DON, since more than 20% reduction was seen. Moreover, the 90% CI of the ratio of log(AUC_0–∞_) was 54–88% and therefore almost completely fell outside the range 80–125%, showing a significant effect of the detoxifier in reduction of systemic exposure to DON.

**Table 1:** Toxicokinetic (TK) parameters of the metabolite DON-S after oral administration of DON alone (0.5 mg/kg BW) and DON in combination with the detoxifier (0.4 g/kg BW, inclusion rate of 4 kg/t) to 8 broiler chickens.

TK parameter (units)	DON	DON + SEP/MONT/AC	*P* value
AUC_0–∞_(min.response)	4305.39 ± 1684.29	2919.59 ± 1032.94	**0.010**
C_max_ (response)	51.93 ± 21.04	34.39 ± 17.06	**0.013**
T_max_ (min)	64.38 ± 42.71	56.25 ± 38.24	0.637
k_e_ (1/min)	0.013 ± 0.001	0.013 ± 0.002	0.976
T_1/2e_ (min)	53.09 ± 6.09	53.77 ± 9.40	0.877
Relative F AUC_0–∞_ (%)	/	67.81	/
90% CI for log(AUC_0–∞_)	/	[0.54; 0.88]	/

The mean ± standard deviation (SD) is shown.

Significant values are in bold.

AUC_0–∞_ area under the DON-S response-time curve from time 0 to infinity, *C*_*max*_ maximum DON-S response in plasma, *T*_*max*_ time at maximum DON-S plasma response, *Relative F* relative oral bioavailability, *CI* confidence interval, /: not applicable.

## Conclusions

We have demonstrated a machine learning-aided approach to the design of mycotoxin detoxifiers (MDTs). We used a dataset of experimental in vitro assessment of the adsorption and efficiency of various MDTs against regulated mycotoxins (DON, T2, ZEN, OTA, FB1, AFB1) to build two random forest (RF) models which predict the adsorption (RFads) and efficiency (RFeff). The model feature space was defined by descriptors representing MDT and mycotoxin physico-chemical properties and in vitro experimental setting which modelled the animal gastrointestinal tract. The model achieved satisfactory performance with R^2^ of 0.92 and 0.96 and MAE of 5.5% and 4.8% for RFads and RFeff, respectively. By means of feature importance analysis of the models, we gain insights into the MDT mode of action i.e., the unequivocal heavy contribution of Mg^2+^ suggests that the main mechanism processes through substitutions of the exchangeable cations and chelate formations. Our RFeff being skilled to capture the synergy between components MDT formulations was employed in the recognizing of specific material preparation which avoids interferences originated by micronutrient adsorption. The top promising formulation incorporates sepiolite, montomorillonite and activated carbon in ratio 1:4:0.1 (SEP/MONT/AC). Our RFeff targets DON as the most challenging to-be-removed molecule which was trained for the in vivo validation of the real performance of SEP/MONT/AC on the oral bioavailability of DON in broiler chickens. The specifically designed in vivo trial avoids the repetition of pointless use of animals following the 3Rs principles. Both DON and the corresponding phase II metabolite (DON-S) detection demonstrated the relevant reduction in in vivo systemic exposure to DON in broiler chickens, confirming the findings of our approach, i.e., the capability of the identified material to mitigate the presence of the challenging to be removed DON. Our computer-aided approach enables versatile applications, e.g. assisting the recommendation of the precise administration dosage of MDTs for specific contamination levels, and providing the estimation of adsorption efficiency of MDTs against yet-to-be-regulated mycotoxins projecting future assessment of emerging contaminants.

## Methods

### MDT in vitro assessment

The in vitro adsorption experiments employ a specific amount of MDT with the appropriate amount of mycotoxin (ranging between 0.5 and 5 µg/ml) in phosphate buffer adjusted to the required pH (see details in Supplementary Sect. S1). The adsorption is then estimated against the amount of toxin in the standard by the following Eq. (1):

1 $${\text{Ads}}\left( \% \right) = 100{-}\left( {{\text{A}}_{{{\text{ft}}}} {\text{/A}}_{{{\text{st}}}} *100} \right)$$

where A_ft_ and A_st_ are the amount of free toxin, after separating the complexed MDT-mycotoxin and the toxin amount in the standard, respectively. The MDT-mycotoxin complex is then dispersed in buffered aqueous solution of neutral pH, followed by the incubation and centrifugations steps. The desorption is calculated as follow (Eq. (2)):

2 $${\text{Des}}\left( \% \right) = {\text{A}}_{{{\text{rt}}}} {\text{/A}}_{{{\text{st}}}} *100$$

where A_rt_ is the amount of toxin released by the MDT. The efficiency is then calculated as (Eq. (3)):

3 $${\text{Eff}}\left( \% \right) = {\text{Ads}}\left( \% \right){-}{\text{Des}}\left( \% \right)$$

### Datasets, descriptors, and target outcomes

The dataset of in vitro adsorption and desorption experiments contains 51 data points related to 10 natural clays and 33 data points of 5 clay-based composites which incorporate activated charcoal (AC) as organic component. The natural clays represent four morphological diverse classes and were, i.e., 2 sepiolites, 2 swelling montmorillonites, 2 non-swelling montmorillonites, and 4Mg-rich smectites, with high purity (> 80% of phyllosilicate content, the remaining amount are impurity such as carbonates, quartz, and calcites, not considered as factor of interest in our application). The descriptor space was defined by selecting the main features which can efficiently represent MDT materials, mycotoxins and in vitro experimental settings (summarized in Table 2). Clays are identified by experimentally obtained properties (details in Supplementary Sect. S1). Two additional descriptors were included completing the vector space for the recognition of composite materials, i.e., type of AC (0 for inorganic material, 1 for CA with 900 m^2^/g and 2 for AC with 1200 m^2^/g), and its content with respect to dried inorganic component (AC (%)). Mycotoxins were featured by the most common molecular descriptors supplied in PubChem repository and Toxin and Toxin Target Database (T3DB). In vitro experiments were modeled by adsorption and desorption pH, inclusion rate, toxin concentration and the relative amount of MDT with respect to the toxin concentration (MDT/Toxin) details in Supplementary Sect. S1). The targets were the previously defined Ads(%) and Eff(%) outcomes.

**Table 2:** Input vector space.

Mycotoxin molecular descriptor	Detoxifier material descriptors	Experimental settings
Molecular weight (MW)	Specific surface area (BET)	Adsorption pH
Octanol/water partition coefficient (XlogP)	Cation exchange capacity (CEC)	Desorption pH
Hydrogen bond donor (H-don)	pH	Inclusion rate MDT
Hydrogen bond acceptor (H-acc)	Exchangeable cations (Na^+^, Ca^2+^, Mg^2+^)	Toxin concentration
Rotatable bonds (Rot-Bond)	AC	MDT/toxin
Topological polar surface area (TPSA)	AC (%)	
Specific topological polar surface area (TPSA/MW)		
Amide bonds		
Aromatic rings		
π counts		
pKa (strongest acid)		
pKa (strongest basic)		
Physiological charge		
Water solubility		
Polarizability		

### Model architectures

Random forest regressor (RF) was trained using sci-kit learn Python library. The performance, in terms of cross validation score, was assessed against Extra trees regressor (EXT) predicting the detoxification efficiency. Both models predicting Ads(%) and Eff(%) were hyperparameter optimized on the base of k-fold cross validation with K = 5. The optimized hyperparameters as well as the assessment scores are given in Supplementary Tables S2 and S3, respectively. Random forest was selected for its outperforming, low variability within the fold switching and clear model interpretability, exhibiting a R^2^ of 0.84 ± 0.08 in cross-validation of trainset (K = 5) compared to 0.76 ± 0.14 of EXT. The algorithms were trained on 85% of the available dataset and tested in the remaining 15%. Diverse predictive models, i.e., support vector machine, multiple layer perceptron and K-nearest neighbors were benchmarked. The algorithms were assessed by means of R^2^, MEA, accuracy, and p-value, the latter being calculated with the 5 × 2cv paired t test (details available in Supporting Information). The computed p-values allowed to compare RF to the other models to observe a significant difference (*P* < 0.05) in the performances of multiple layer perceptron and K-nearest neighbors and considerable statistical difference between RF and support vector machine. Details are available in Supporting information, Sect. S2, Fig. S8, and Table S4. The models’ codes are available in zenodo repository with the identifier https://doi.org/10.5281/zenodo.5793956.

### Capturing synergy

A number of hybrid formulations were screened for searching of optimal detoxifier material. RF predicted the efficiency allowing to identify the top promising formulation in composite materials composed by SEP, MONT and AC organic compound. A set of diverse formulations was proposed fixing the inorganic portion to SEP/MONT 1:4 and varying the AC from 0 to 5% with respect to the composite weight. Each material descriptor value of the proposed formulations was linearly calculated as $\sum\nolimits_{{\text{i}}} {{\text{a}}_{{\text{i}}} {\text{x}}_{{\text{i}}} }$, where a_i_ is the amount (%) of the i-component in the hybrid formulations and x_i_ is the descriptor value corresponding to the pure i-component.

RF predicted the efficiency of the hypothetical composites (ypred) against the removal of deoxynivalenol (DON), T2-toxin (T2), ochratoxin A (OTA), zearalenone (ZEN), fumonisin B1 (FB1), aflatoxin B1 (AFB1). We compared the prediction with the linear combination of efficiency of the pure components, being calculated as $\sum\nolimits_{{\text{i}}} {{\text{a}}_{{\text{i}}} {\text{y}}_{{\text{i}}} }$, where y_i_ is the predicted target of the pure i-component. The gap between predictions and linear combination of pristine efficiency was assigned to the synergistic effect between components (Eq. (4)), being it positive or negative depending on the specific binder-toxin affinity.

4 $${\text{Synergy}} = y_{pred} - \sum\limits_{{\text{i}}} {{\text{a}}_{{\text{i}}} {\text{y}}_{{\text{i}}} }$$

### In vivo validation

An in vivo trial was conducted with 8 healthy broiler chickens, females and males equally divided and about the same body weight (BW) at arrival (see supporting information, Supplementary Sect. S3 for details). The in vivo study was conducted at CER-Groupe, a GLP (Good Laboratory Practice) compliant test site (Marloie, Belgium). The animal study was approved by the Ethical Committee of CER-Groupe (approval number CE/Sante/Residus-1). The animal experiment was conducted in compliance with Council Directive No. 2010/63/EU of 22 September 2010 on the protection of animals used for scientific purposes^44^ as well as the ARRIVE guidelines. Randomization was performed at arrival, based on the sex and BW of the broiler chickens in such a way that 2 groups with 4 birds (2 males and 2 females) each were formed with about the same average BW/group. The feed was analyzed before administration by a multi-mycotoxin LC–MS/MS method (liquid chromatography-tandem mass spectrometry) and was found to contain low levels of DON (61.3 µg/kg) and OTA (1.6 µg/kg). These contamination levels were well within the acceptance criteria of the EU (2006/576/EC)^2^. The treatment consisted of a single oral bolus administration with either DON or DON in combination with SEP/MONT/AC detoxifier (0.500 mg DON/kg BW, corresponding to the maximum EU guidance level of 5 mg/kg DON in feed, and 0.4 g detoxifier/kg BW, corresponding to an inclusion rate of 4 kg/t in the feed), administered as oral capsules directly in the crop and using a cross-over study design respecting a one-day wash-out period between treatments. Repetitive blood samples were taken from the *vena metatarsalis plantaris superficialis* (leg vein). The time points of blood sampling were 0 h (just before administration) and 0.08, 0.25, 0.5, 0.75, 1, 1.5, 2, 4, and 8 h (post administration, p.a.). The blood samples were centrifuged within 2 h after collection. Both DON and its major phase II metabolite DON-sulphate (DON-S) were analysed as appropriate biomarkers for exposure by UHPLC-MS/MS^45^. For DON-S, a qualitative UHPLC-MS/MS analysis was performed, i.e. chromatographic response or peak area ratio of DON-S/internal standard. Toxicokinetic modeling of the chromatographic response-time profiles of DON-S was done by non-compartmental toxicokinetic analysis (details in Supplementary Sect. S3). Following parameters were calculated: area under the response-time curve from time zero to infinite (AUC_0–∞_), maximal DON-S response in plasma (C_max_), time at maximal plasma response (T_max_), elimination half-life (T_1/2e_) and elimination rate constant (k_e_). The effect of the detoxifier on the oral absorption of DON was evaluated by comparing major toxicokinetic parameters between the DON and DON + detoxifier treated broiler chickens, with special emphasis on AUC_0–∞_, Cmax and Tmax. Statistical analysis was performed with to evaluate possible significant differences between the DON and DON + detoxifier treatment. *P* values < 0.05 were considered significant. Moreover, the relative oral bioavailability ((AUC_0–∞_ mycotoxin + detoxifier/AUC_0–∞_ mycotoxin)*100) was evaluated as marker for efficacy of the detoxifier. In general, two treatments are considered bioequivalent or thus not different from one another if the 90% confidence interval (CI) of the ratio of a log-transformed exposure measure (AUC) falls completely within the range 80–125%, as it is assumed that differences in exposure up to 20% are not relevant. If the CI falls completely out this specified range the treatments are considered not bioequivalent, and hence a significant effect of the detoxifier in reduction of systemic exposure can be concluded.

## Supplementary Information

Supplementary Information.

## Data Availability

The dataset and the model codes that support the findings of this study are available in zenodo repository with the identifier https://doi.org/10.5281/zenodo.5793956.
