# Supplementary material for: Machine learning-aided design of composite mycotoxin detoxifier material for animal feed
Source: Sci Rep. 2022 Mar 22;12:4838. doi: 10.1038/s41598-022-08410-x (PMC8941095; doi:10.1038/s41598-022-08410-x)
Supplement: Supplementary file 1 — Supplementary Information. [file 41598_2022_8410_MOESM1_ESM.pdf]

Supporting information

Machine learning-aided design of composite mycotoxin detoxifier material for animal feed

Giulia Lo Dico,[a,b,c], Siska Croubels[d], Verónica Carcelén[c] and Maciej Haranczyk[a]

[a]IMDEA Materials Institute, C/Eric Kandel 2, 28906 Getafe, Madrid, Spain.
[b]Department of Materials Science and Engineering, Universidad Carlos III de Madrid, Avda. de la Universidad, 30. 28911 Leganés, Madrid, Spain.
[c]Tolsa Group, Carretera de Madrid a Rivas Jarama, 35, 28041, Madrid, Spain.
[d]Department of Pathobiology, Pharmacology and Zoological Medicine, Faculty of Veterinary Medicine, Ghent University, Salisburylaan 133, 9820, Merelbeke, Belgium.

Additional figures and tables accompanying the main text.

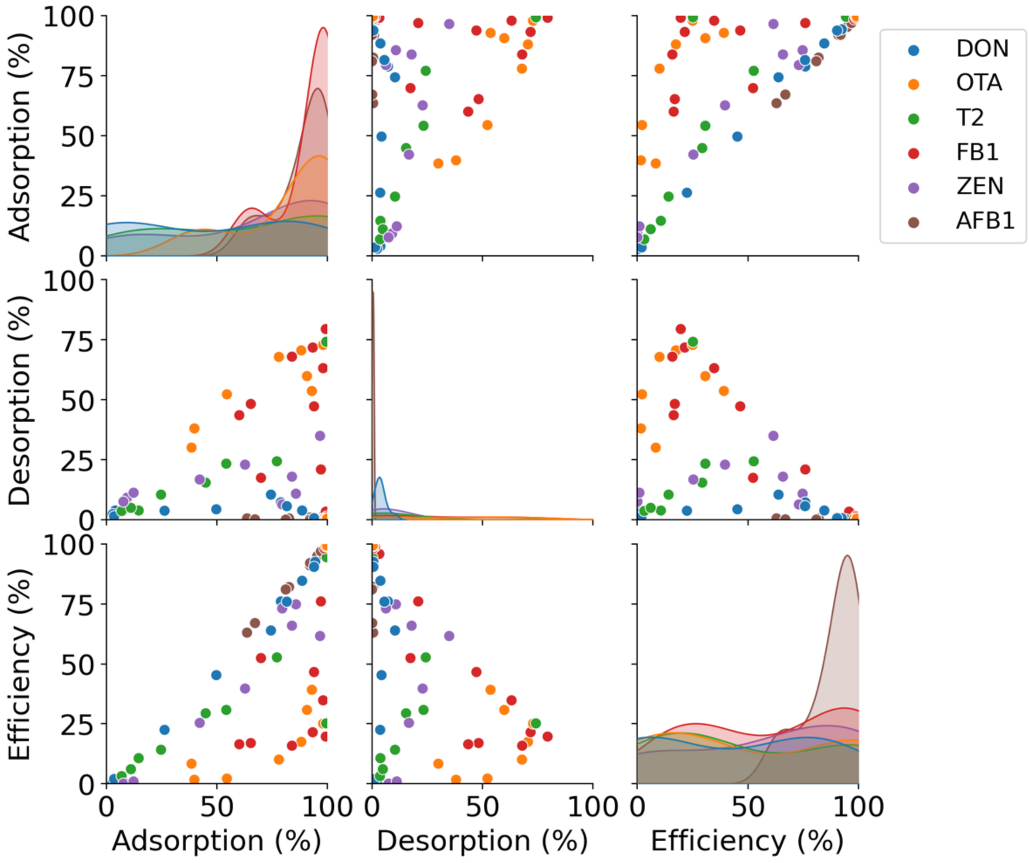

Figure S1. Pairwise relationships of adsorption, desorption, and efficiency in the dataset.

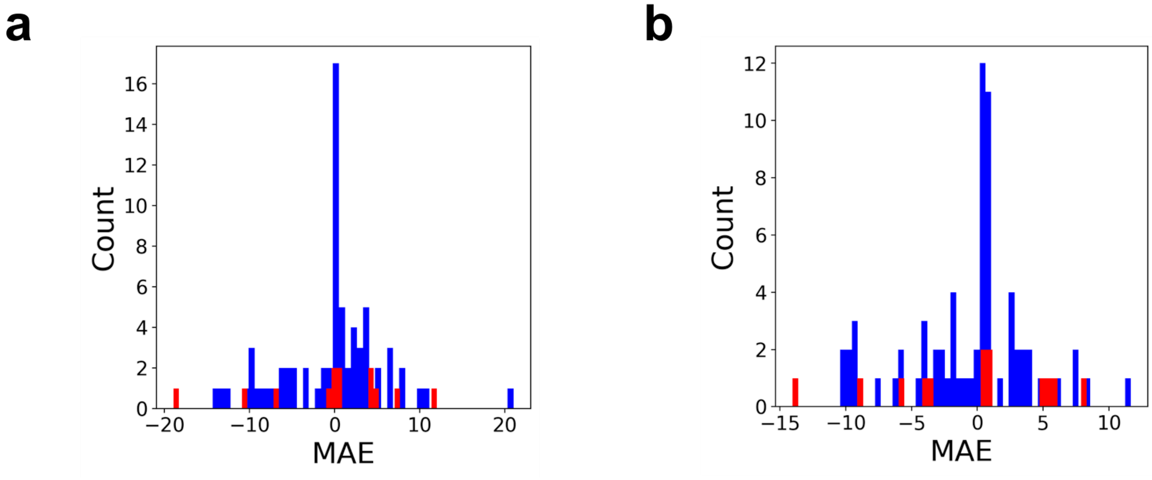

Figure S2. Mean absolute error distribution corresponding to the model predicting the adsorption (A) and efficiency (B).

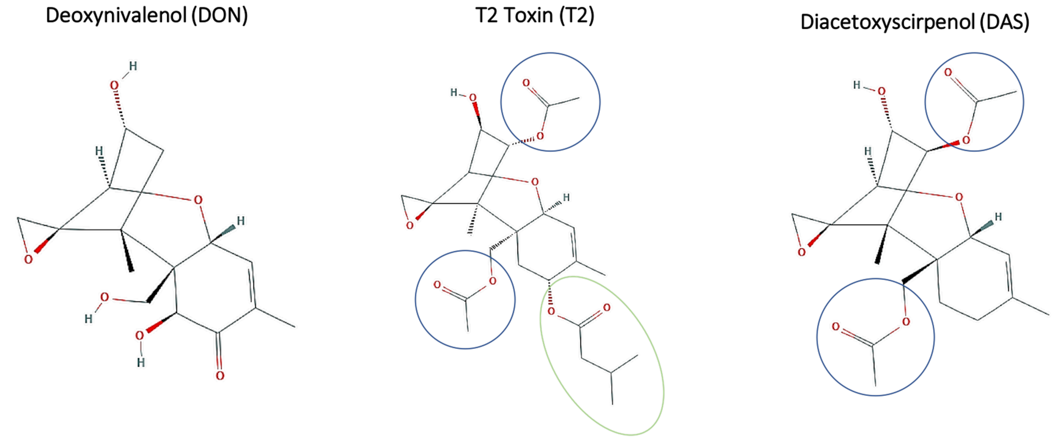

Figure S3. Chemical structures of three *Fusarium* toxins deoxynivalenol (DON), T-2 toxin (T2), and diacetoxyscirpenol (DAS). Blue circles highlight the carboxy groups, shared between T2 and DAS and absented in DON. Green circles highlight the methylbutanoate group present uniquely in T2 structure.

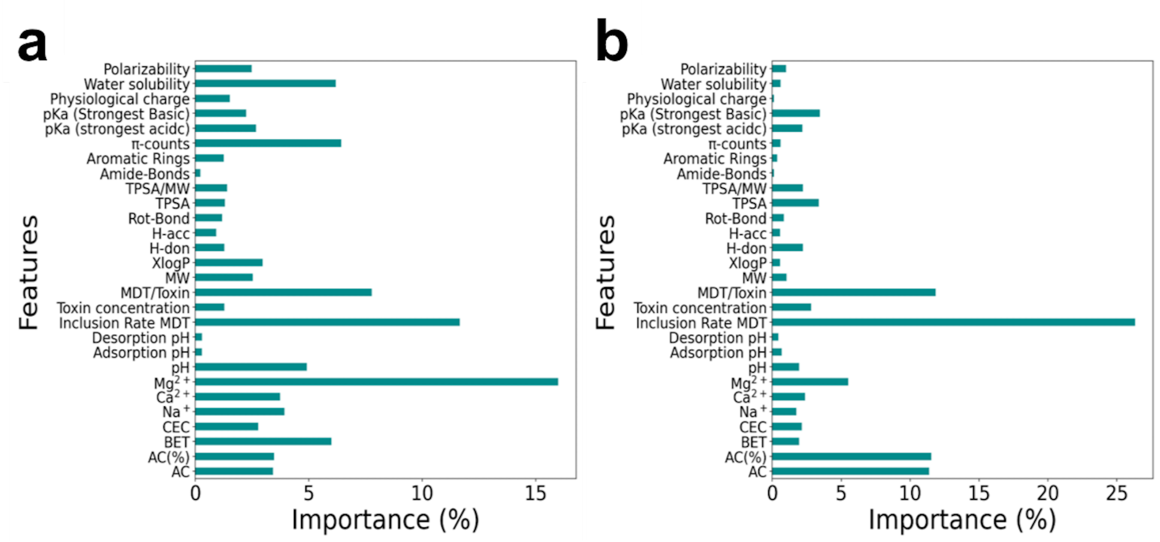

Figure S4. Feature importance score extracted by RFads (A) and RFeff (B).

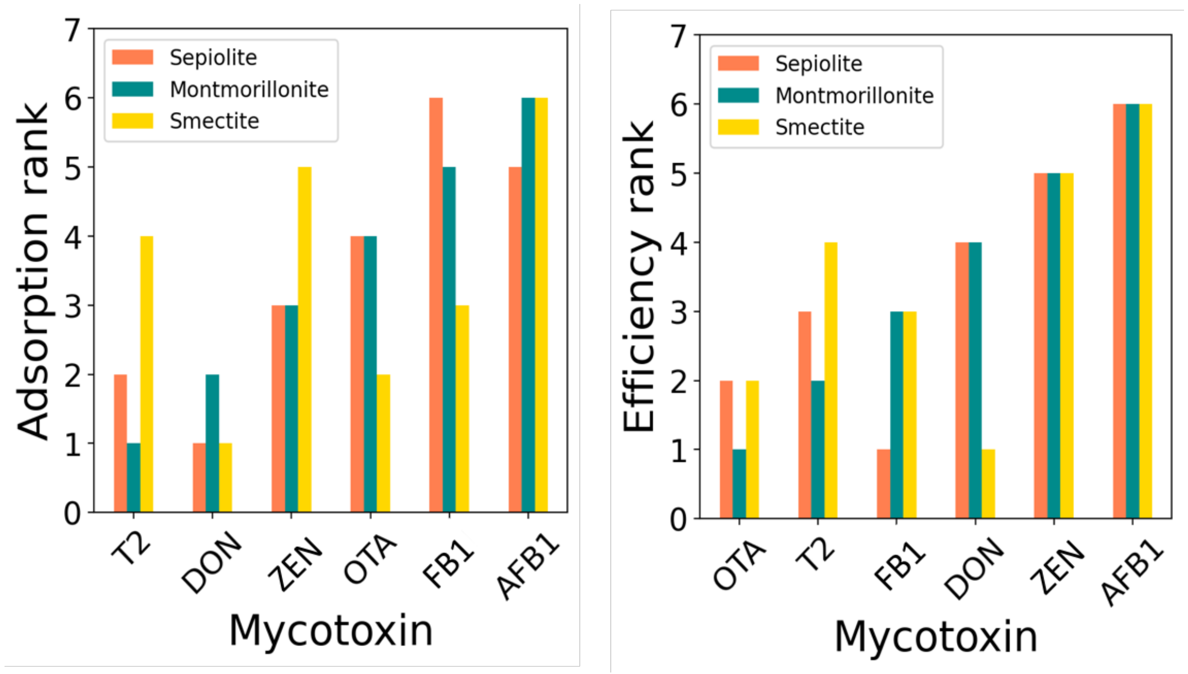

Figure S5. Performance ranking of sepiolite, Na-montmorillonite and Mg-rich smectite in terms of adsorption and efficiency. The experimental settings for the uptake of DON, OTA, T2, FUM and ZEN were fixed to 1kg/t of inclusion rate of MDT, 3 µg/ml of toxin concentration. The pH during the adsorption experiment was fixed to 3 while the desorption pH was 6.5. AFB1 uptake was assessed with 2kg/t of inclusion rate of MDT, 4 µg/ml of toxin concentration. The pH during both adsorption and desorption experiments was fixed to 5.

Table S1. The list of mycotoxins employed in the computer-aided screening of hybrid materials towards multi-mycotoxin uptake.

**Toxin**	**Cod.**	**Fungi species**	**Mycotoxin group**
Deoxynivalenol	DON	*Fusarium* species	Trichothecenes
15-acetyl-deoxynivalenol	15ADON	*Fusarium* species	Trichothecenes
3-acetyl-deoxynivalenol	3ADON	*Fusarium* species	Trichothecenes
T2-toxin	T2	*Fusarium* species	Trichothecenes
T2-toxin tetraol	T2T	*Fusarium* species	Trichothecenes
HT2-toxin	HT2	*Fusarium* species	Trichothecenes
Diacetoxyscirpenol	DAS	*Fusarium* species	Trichothecenes
Aflatoxin B1	AFB1	*Aspergillus* species	Aflatoxins
Aflatoxin B2	AFB2	*Aspergillus* species	Aflatoxins
Aflatoxin G1	AFG1	*Aspergillus* species	Aflatoxins
Aflatoxin G2	AFG2	*Aspergillus* species	Aflatoxins
Aflatoxin M1	AFM1	*Aspergillus* species	Aflatoxins
Aflatoxin M2	AFM2	*Aspergillus* species	Aflatoxins
Fumonisin B1	FB1	*Fusarium* species	Fumonisins
Fumonisin B2	FB2	*Fusarium* species	Fumonisins
Fumonisin B3	FB3	*Fusarium* species	Fumonisins
Zearalenone	ZEN	*Fusarium* species	Zearalenone
alfa-zearalenol	AZEL	*Fusarium* species	Zearalenone
beta-zearalenol	BZEL	*Fusarium* species	Zearalenone
Ochratoxin A	OTA	*Aspergillus* species	Ochratoxins
Ochratoxin B	OTB	*Aspergillus* species	Ochratoxins
Ochratoxin C	OTC	*Aspergillus* species	Ochratoxins

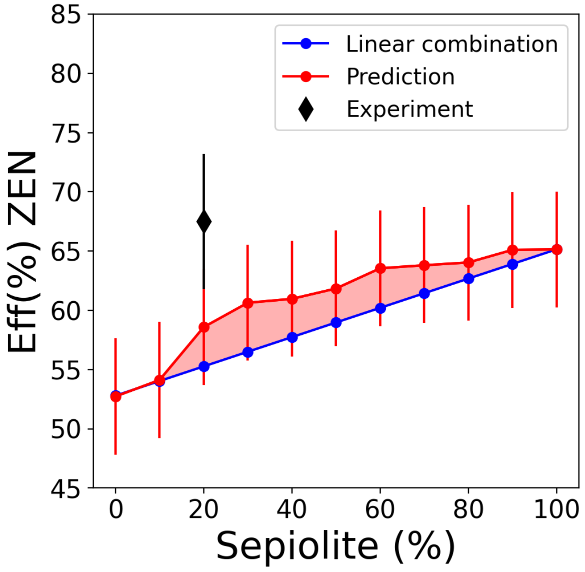

Figure S6. RF-based synergy-capturing assessment of efficiency in a series of hybrid SEP/MONT materials. The red area is assigned to the positive synergistic effect. The experimental settings for the uptake of DON, OTA, T2, FB1 and ZEN were fixed to 2 kg/t of inclusion rate of MDT, 2 μg/ml of toxin concentration. The pH during the adsorption experiment was fixed to 3 while the desorption pH was 6.5. The main values and the MAE are given.

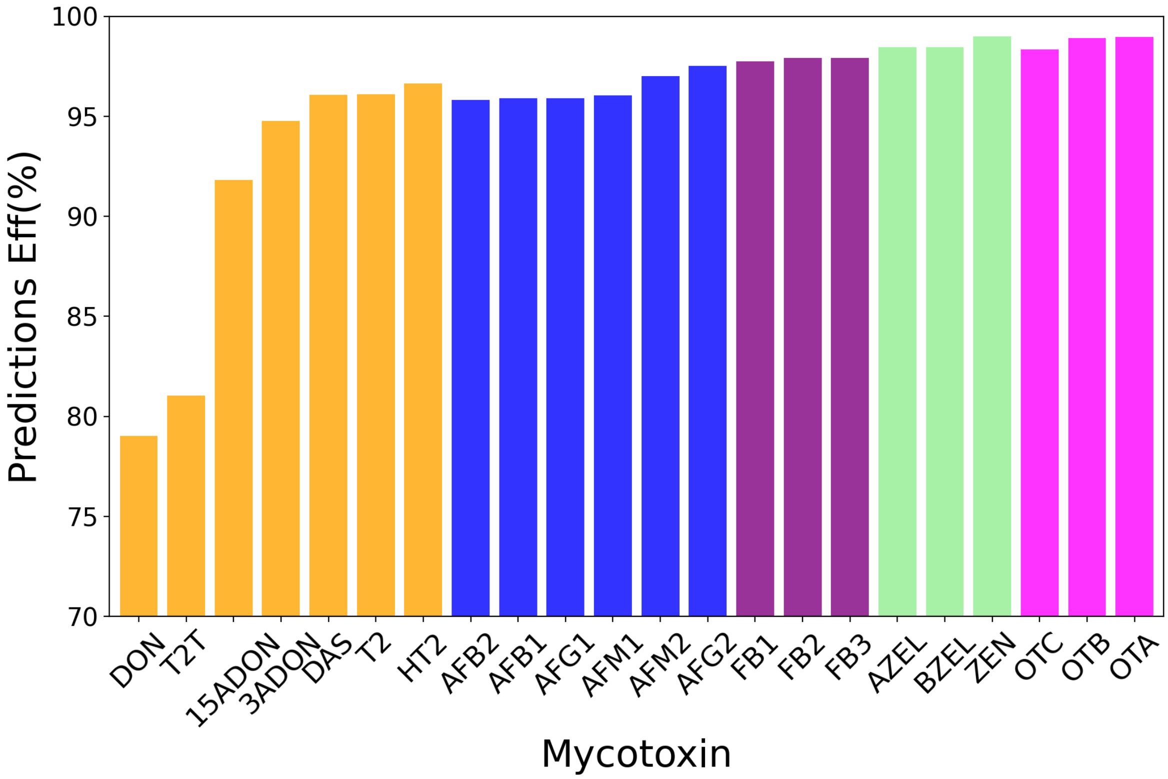

Figure S7. Efficiency prediction of a list of regulated and yet un-regulated mycotoxins. In orange the group of trichothecenes, aflatoxins in blue, fumonisins in purple, zearalenone in green and ochratoxins in magenta. The experimental setting was fixed to 2kg/t of inclusion rate of MDT, 2 µg/ml of toxin concentration. The pH during the adsorption experiment was fixed to 3 while the desorption pH was 6.5.

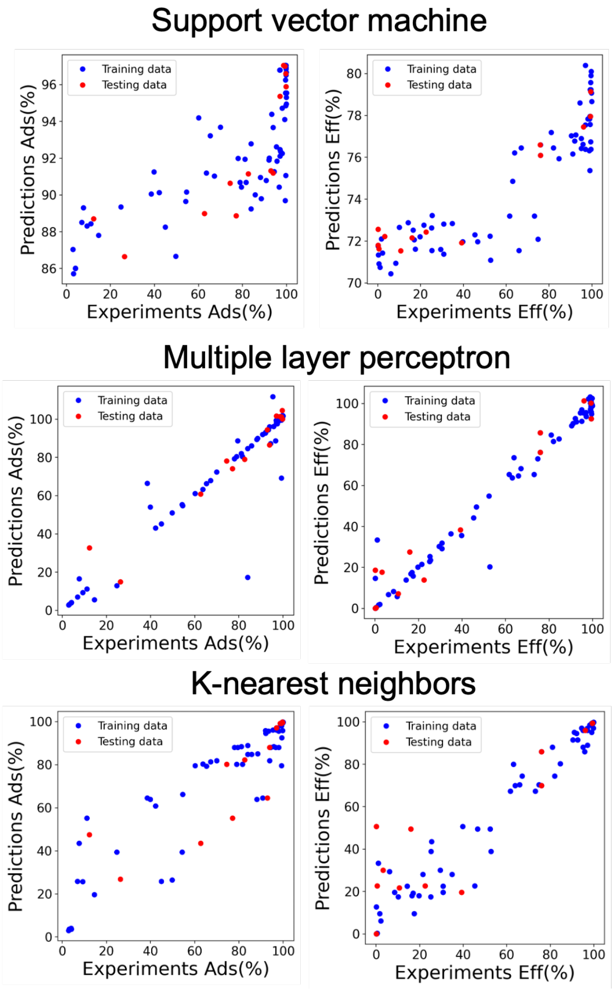

Figure S8. Graphical assessment of support vector machine, multiple layer perceptron and k-nearest neighbors predicting Ads(%) and Eff(%).

Table S2. Optimized hyperparameters of RFads and RFeff after cross-validation with K=5.

Hyperparameter	RFads	RFeff
n_estimators	2000	2000
max_depth	300	300
max_features	9	12
min_samples_leaf	1	1
min_samples_split	2	2

Table S3. Assessment scores of RFads and RFeff.

Assessment	RFads	RFeff
R^2 (testset)	0.91	0.97
R^2 (trainset, K=5)	0.71±0.054	0.84±0.08
MAE (testset)	5.5	4.8
MSE (testset)	60	38

Table S4. Model assessment of support vector machine, multiple layer perceptron y k-nearest neighbors and Random forest regressor predicting Ads(%) and Eff(%).

Score	Support vector machine	Multiple layer perceptron	K-nearest neighbors	Random forest regressor
Target		Ads(%)		
p-value	0.284	<0.0005	0.007	
R^2	0.66	0.87	0.7	0.91
MAE	12.6	7	9.1	5.5
Accuracy	0.39	0.46	0.54	0.69
Target		Eff(%)		
p-value	0.372	<0.0005	0.007	
R^2	0.84	0.95	0.73	0.97
MAE	10.34	6.2	13.9	4.8
Accuracy	0.46	0.69	0.38	0.69

[a] The p-value is calculated by 5x2cv paired t-test comparing each model-B (Support vector machine, multiple layer perceptron and k-nearest neighbors) against Random Forest regressor. The null hypothesis is that Random Forest and model-B have equal performance (α = 0.05).

S1. Experimental material characterization

Specific surface area was estimated by Brunauer–Emmett–Teller (BET) theory on the adsorption branch of the physisorption isotherms. The isotherms were measured in a Micromeritics Gemini V surface area and pore size analyzer under N_2 gas flow at 77K. The samples were previously degassed at 124°C for 18h. The pH of a 10 wt% suspension of raw clay in distilled water was measured with Crison Basic 20 pH-meter, after stirring for 10 minutes at 25°C. The cation exchange capacity (CEC) was estimated quantifying the concentration of colored Cu^{2+}-triethylenetetramine complex by spectrophotometry. The exchange solution (1 l) was prepared dissolving in deionized water 1.45 ml of triethylenetetramine (97 %, Sigma-Aldrich) and 100 ml of copper sulfate standard solution 0.1 M (Sigma Aldrich). The exchange solution (30 ml) was added to 0.15g of clay sample stirring vigorously the suspension by vortex agitator until there are no more visible agglomerations detectable. The suspension was then centrifuged for 5 min at 4.500 min^{-1} and supernatant solution measured using a UV spectrophotometer (Agilent Technologies, Cary 60 UV-vis) at 580 nm. The cations leached by the clay structure (Na^+, Ca^{2+}, Mg^{+2}), in the supernatant solution, were evaluated by chemical analysis with ICP-OES Varian, Agilent 730.

The *in vitro* experiments are performed by Trilogy laboratory validated method modelling the adsorption and desorption through the gastrointestinal tract. Firstly, a specific amount of binder

with the appropriate amount of mycotoxin were combined in phosphate buffers adjusted to the required pH, incubating the mixture at 37°C for 3 h under gently stirring. The solution was then centrifugated at 8 RPM separating the complexed binder-mycotoxin from the free toxin which is analyzed by HPLC-UV or HPLC-Fluorescence. The adsorption is calculated against the amount of toxin in the standard with the following equation 1:

$$Ads(\%) = 100 - (A_{ft}/A_{st} * 100) \quad \text{Eq. 1}$$

Where A_{ft} and A_{st} are the amount of toxin in the supernatant solution and in the standard, respectively. The separated binder-mycotoxin complex was then taken through a desorption step. A buffered aqueous solution of neutral pH is added to the complex keeping the solution under stirring at 37°C for 3 hours. After incubation, the solution was centrifugated removing the remaining binder-mycotoxin complex from any toxin released from the binder. The supernatant was the analyzed by HPLC-UV or HPLC-Fluorescence and the desorption calculated as follow (Eq.2):

$$Des(\%) = A_{rt}/A_{st}*100 \quad \text{Eq. 2}$$

Where A_{rt} is the amount of toxin released by the binder. The efficiency is then calculated as:

$$Eff(\%) = Ads(\%) - Des(\%) \quad \text{Eq. 3}$$

The starting amount of mycotoxin (toxin concentration) and binder (inclusion rate) used for filling the databases, as well as the adsorption and desorption pH are selected among the ranges shown in Table S5.

Table S5. Experimental setting implemented to test the *in vitro* adsorption and desorption of any mycotoxin-binder contained in the databases.

Parameter	Ranges
Adsorption pH	3-5
Desorption pH	6-7
Inclusion Rate (kg/t)	1-4
Toxin Concentration (µg/ml)	0.5-5

S2. Non-tree models architecture

Support vector machine and k-nearest neighbors were implemented using Scikit-learn library. Support vector machine was optimized on the base of k-fold cross validation finding the best hyperparameter, i.e., kernel=’poly’, gamma=’scale’, degree=3. Multiple layer perceptron (MLP) was implemented in TensorFlow and Keras framework. The network was built with 6 dense layers with ReLU activation function, 100 neurons in the hidden layers, Adaptive Moment Estimation (Adam) as optimizer and 300 epochs. The performances were evaluated by R^2 score, mean absolute value (MAE), p-value and accuracy score. The latter is added to classify how often our regressors make predictions closed to the real value by considering the estimated experimental error as tolerance grade. The accuracy is defined as ratio of number of correct prediction and the total predictions corresponding to the testset size. The correct prediction was defined considering

the estimated experimental error (5.7) and obtained when the absolute value of the difference between the predicted and real target is less the 5.7.

S3. *In vivo* trials.

An *in vivo* trial was conducted with 8 healthy broiler chickens, females and males equally divided, of the same breed (Ross 308, commercial supplier, Aye, Belgium), the same age (4 weeks at the beginning of the treatment) and about the same body weight (BW) at arrival. The *in vivo* study was conducted at CER-Groupe, a GLP (Good Laboratory Practice) compliant test site (Marloie, Belgium). The animal study was approved by the Ethical Committee of CER-Groupe. Randomization was performed at arrival, based on the sex and BW of the broiler chickens in such a way that 2 groups with 4 birds (2 males and 2 females) each were formed with about the same average BW/group. Both groups were housed in the same pen of 3 m². The housing conditions were according to the EU guidelines and the Belgian guidelines[1,2]. The light schedule was a 18h/6h light/dark scheme. An acclimatization period of 8 days was respected (day 1-8). Throughout the acclimatization period, the chickens were allowed access to feed and water *ad libitum*. The chickens received commercial broiler chicken feed ACTI POUSSIN (batch 028590, SCAR Büllingen, Belgium). At least eight hours before the treatment (day 8), the feed was withdrawn, but water was available. Animals were fed again 4 h post administration. The feed was analysed by a multi-mycotoxin LC-MS/MS method (liquid chromatography-tandem mass spectrometry) and was found to contain low levels of DON (61.3 µg/kg) and OTA (1.6 µg/kg). These contamination levels were well within the acceptance criteria of the EU (2006/576/EC)[3]. Analytical standard of DON was obtained from Fermentek (Jerusalem, Israel), and a stock solution was prepared in ethanol at a concentration of 10 mg/ml.

The treatment consisted of a single oral bolus administration with either DON or DON in combination with SEP/MONT/AC detoxifier (0.500 mg DON/kg BW, corresponding to the maximum EU guidance level of 5 mg/kg DON in feed, and 0.4 g detoxifier/kg BW, corresponding to an inclusion rate of 0.4% in the feed), administered as oral capsules directly in the crop and using a cross-over study design respecting a one-day wash-out period between treatments. The capsules contained cellulose and the appropriate volume of DON stock solution was added, whether or not combined with the detoxifier. Capsules were closed after 20 min in order to ensure evaporation of the solvent.

Repetitive blood samples (+/- 0.5 ml) were taken from the *vena metatarsalis plantaris superficialis* (leg vein). Intravenous (IV) catheters were placed in order to ensure continuous access to the blood vein. The time points of blood sampling were 0 h (just before administration) and 0.08, 0.25, 0.5, 0.75, 1, 1.5, 2, 4, and 8 h (post administration, p.a.). The blood samples were centrifuged within 2 hours after collection (±3,000 *g*, 10 min, 4°C). Plasma was stored at ≤-15°C until shipment to the Laboratory of Pharmacology and Toxicology for GLP-compliant analysis by UHPLC-MS/MS. Both DON and its major phase II metabolite DON-sulphate (DON-S) were analysed as appropriate biomarkers for exposure[4]. For DON-S, a qualitative UHPLC-MS/MS analysis was performed, i.e. chromatographic response or peak area ratio of DON-S/internal standard.

Toxicokinetic modeling of the chromatographic response-time profiles of DON-S was done by non-compartmental toxicokinetic analysis (WinNonlin 6.3, Pharsight Corporation, USA). Following parameters were calculated: area under the response-time curve from time zero to infinite ($AUC_{0-\infty}$), maximal DON-S response in plasma (C_{max}), time at maximal plasma response (T_{max}), elimination half-life ($T_{1/2e}$) and elimination rate constant (k_e). The effect of the detoxifier on the oral absorption of DON was evaluated by comparing major toxicokinetic parameters between the DON and

DON+detoxifier treated broiler chickens, with special emphasis on $AUC_{0-\infty}$, Cmax and Tmax. Statistical analysis was performed with SPSS Statistics 26 to evaluate possible significant differences between the DON and DON+detoxifier treatment. P-values < 0.05 were considered significant.
First, a Shapiro-Wilk test for normality was performed, including inspection of QQ-plots. If the data were not normally distributed, a log-transformation of the data was performed. If OK, a paired sample t-test was performed. Data for elimination half-life ($T_{1/2e}$) were not normally distributed, even after log-transformation. Therefore, a nonparametric related-samples Wilcoxon signed rank test was performed.
Moreover, the relative oral bioavailability (($AUC_{0-\infty}$ mycotoxin+detoxifier/ $AUC_{0-\infty}$ mycotoxin)*100) was evaluated as marker for efficacy of the detoxifier. In general, two treatments are considered bioequivalent or thus not different from one another if the 90% confidence interval (CI) of the ratio of a log-transformed exposure measure (AUC) falls completely within the range 80-125%, as it is assumed that differences in exposure up to 20% are not relevant. If the CI falls completely out this specified range the treatments are considered not bioequivalent, and hence a significant effect of the detoxifier in reduction of systemic exposure can be concluded.

References

(1) European Union. Commission Recommendation on Guidelines for the Accommodation and Care for Animals Used for Experimental and Other Scientific Purposes. *Off. J. Eur. Union* **2010**, *L276/33*.

(2) K.B. van 16 Januari 2006 Tot Vaststelling van de Nadere Regels van de Erkenningen, Toelatingen En Voorafgaande Registraties Afgeleverd Door Het Federaal Agentschap Voor de Veiligheid van de Voedselketen. Belgisch Staatsblad 2006.

(3) European Union. Commission Recommendation 2006/579/EC of 17 August 2006 on the Presence of Deoxynivalenol, Zearalenone, Ochratoxin A, T-2 and HT-2 and Fumonisins in Products Intended for Animal Feeding. *Off. J. Eur. Union L 229/7* **2006**, *L 229/7*, 7–9.

(4) Lauwers, M.; De Baere, S.; Letor, B.; Rychlik, M.; Croubels, S.; Devreese, M. Multi LC-MS/MS and LC-HRMS Methods for Determination of 24 Mycotoxins Including Major Matrices from Pigs and Broiler Chickens. *Toxin* **2019**, *11* (3), 171–201. https://doi.org/10.3390/toxins11030171.
